# From bench to bedside: translating mesenchymal stem cell therapies through preclinical and clinical evidence

**DOI:** 10.3389/fbioe.2025.1639439

**Published:** 2025-07-30

**Authors:** Jai Chand Patel, Meenakshi Shukla, Manish Shukla

**Affiliations:** ^1^ Department of Genetics, Cell Biology and Anatomy, University of Nebraska Medical Center, Nebraska, NE, United States; ^2^ Department of Neurosurgery, Translational and Vascular Research Group, Penn State Milton S. Hershey Medical Center, Hershey, PA, United States

**Keywords:** mesenchymal stem cells (MSCs), paracrine signaling, immunomodulation, regulatory, clinical evidence, regenerative medicine

## Abstract

Mesenchymal stem cells (MSCs) are emerging as a powerful tool in regenerative medicine due to their ability to differentiate into mesenchymal lineages, such as bone, cartilage, and fat, along with their low immunogenicity and strong immunomodulatory properties. Unlike traditional cell therapies that rely on engraftment, MSCs primarily function through paracrine signaling—secreting bioactive molecules like vascular endothelial growth factor (VEGF), transforming growth factor-beta (TGF-β), and exosomes. These factors contribute to tissue repair, promote angiogenesis, and modulate immune responses in damaged or inflamed tissues. Recent studies have identified mitochondrial transfer as a novel therapeutic mechanism, where MSCs donate mitochondria to injured cells, restoring their bioenergetic function. This has expanded the therapeutic potential of MSCs to include conditions such as acute respiratory distress syndrome (ARDS) and myocardial ischemia. Clinically, MSCs have shown efficacy in diseases like graft-versus-host disease (GVHD), Crohn’s disease, and COVID-19. Trials such as REMODEL and REMEDY have demonstrated improved clinical outcomes, further validating MSC-based interventions. However, several challenges remain, including variability in cell potency, poor engraftment, and inconsistent results across clinical trials. Advances in genetic engineering such as CRISPR-modified MSCs and biomaterial scaffolds are being developed to enhance therapeutic efficacy and cell survival. Additionally, AI-driven platforms are being utilized to personalize MSC therapy and optimize cell selection. Innovative approaches like 3D bioprinting and scalable manufacturing are paving the way for more consistent and precise therapies. Moving forward, the integration of mechanistic insights with robust quality control and regulatory frameworks essential to translating MSC therapies from bench to bedside and ensuring their reliable application in clinical practice.

## 1 Introduction: mesenchymal stem cells and their therapeutic potential

**FIGURE 1:**
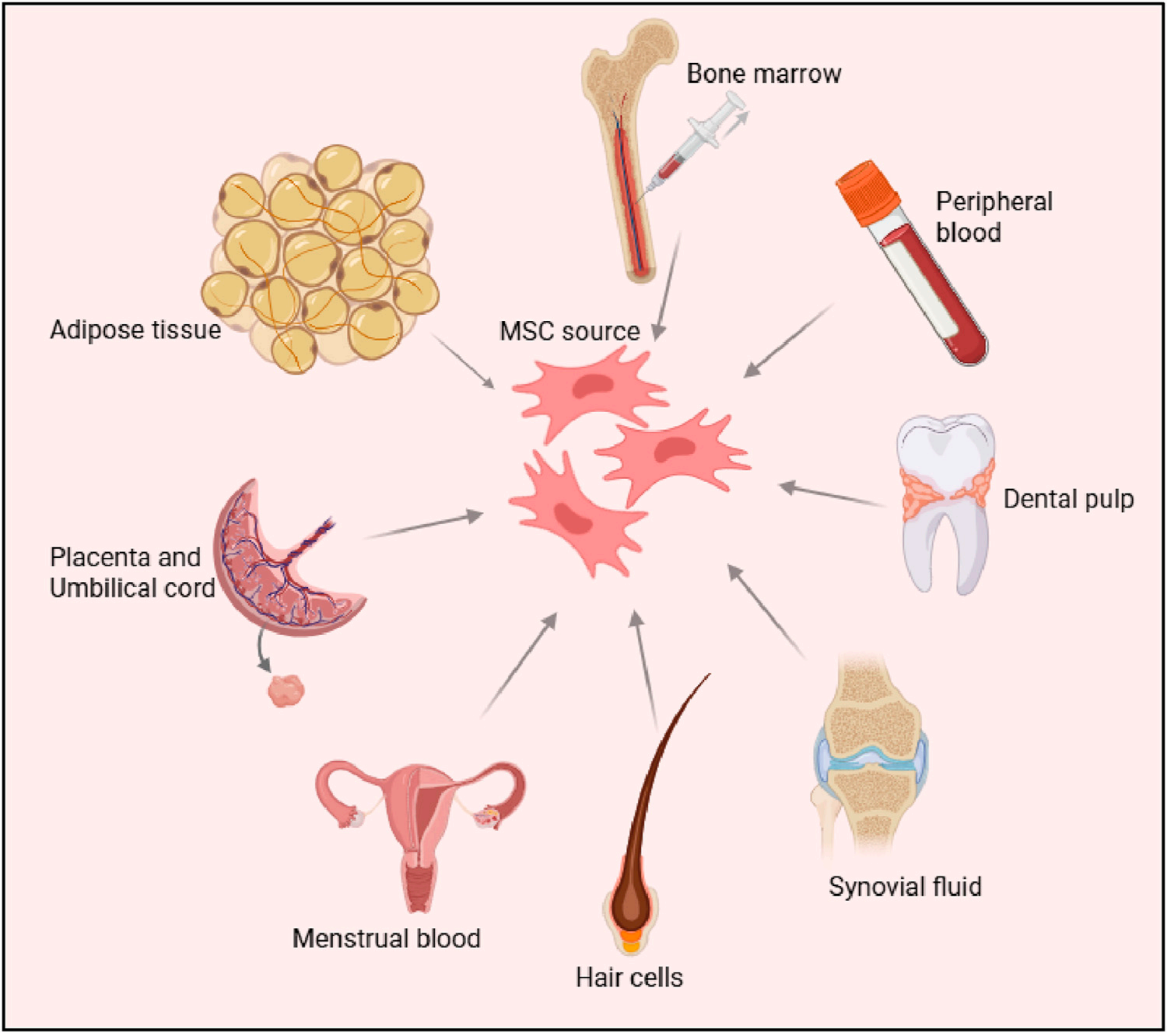
Sources of Mesenchymal Stem Cells (MSCs) The figure illustrates various tissue sources for deriving MSCs, including bone marrow, peripheral blood, adipose tissue, dental pulp, placenta, umbilical cord, synovial fluid, menstrual blood and hair follicle cells. These sources highlight the diverse origins of MSCs, which are widely studied for their regenerative and therapeutic potential.

Mesenchymal stem cells (MSCs) were first identified in the 1970s by Friedenstein and colleagues, who isolated them from bone marrow aspirates (Fridenshtein et al., 1969). These cells stood out due to their ability to adhere to plastic surfaces and form fibroblast-like colonies, distinguishing them from other bone marrow-derived cells (Friedenstein et al., 1970). Since their discovery, MSCs have been found in a wide variety of postnatal tissues, including adipose tissue, umbilical cord (Gronthos et al., 2001), dental pulp (Kadar et al., 2009), synovial fluid (Morito et al., 2008), menstrual blood (Chen et al., 2019), and hair follicles (Wang et al., 2020). Among these sources, adipose-derived MSCs (AD-MSCs) (Gronthos et al., 2001) and umbilical cord-derived MSCs (UC-MSCs), have garnered particular interest due to their abundance and ease of isolation. Hair follicle-derived MSCs (HF-MSCs), obtained from the dermal papilla or sheath, also represent an accessible and promising source due to their multipotent differentiation capacity and regenerative potential (Las Heras et al., 2022). Figure 1.

Unlike pluripotent stem cells such as embryonic stem cells (ESCs) and induced pluripotent stem cells (iPSCs), MSCs are classified as multipotent (Sensebe et al., 2010). This means they can differentiate into several mesenchymal lineages including osteoblasts, chondrocytes, and adipocytes but they do not share the broader differentiation potential of pluripotent cells.

To promote standardization across research studies, the International Society for Cellular Therapy (ISCT) established minimal criteria to define MSCs. These include: (a) adherence to plastic when cultured under standard conditions; (b) expression of specific surface markers CD73, CD90, and CD105 alongside the absence of hematopoietic markers such as CD34, CD45, and CD14; and (c) the ability to differentiate into osteogenic, chondrogenic, and adipogenic lineages *in vitro* (Dominici et al., 2006). One of the defining immunological features of MSCs is their lack of major histocompatibility complex class II (MHC-II) expression, which reduces their immunogenicity (Machado Cde et al., 2013). This low immunogenic profile supports their use in allogeneic settings, allowing for “off-the-shelf” therapeutic applications without the need for strict HLA matching. As a result, MSCs have become highly attractive for regenerative medicine and cell-based therapies (Kot et al., 2019).

### 1.1 Mechanisms Underlying MSC therapeutic effects

Mesenchymal stem cells (MSCs) exert their therapeutic effects primarily through two mechanisms: direct differentiation into tissue-specific cell types and paracrine signaling via the secretion of bioactive molecules (Bagno et al., 2022; Han et al., 2022). Although early research focused heavily on their ability to differentiate, more recent findings emphasize that the predominant therapeutic impact of MSCs arises from their paracrine activity (Baglio et al., 2015). This includes the release of extracellular vesicles (EVs), cytokines, and growth factors that influence surrounding cells and tissues (Chen et al., 2008).

In terms of immunomodulation, MSCs interact with both innate and adaptive immune systems to help restore immune balance. They inhibit T-cell proliferation through the secretion of immunosuppressive agents such as prostaglandin E2 (PGE2), indoleamine 2,3-dioxygenase (IDO), and programmed death-ligand 1 (PD-L1), thereby tempering overactive immune responses (Glennie et al., 2005; Selmani et al., 2008). Moreover, MSCs guide macrophage polarization by converting pro-inflammatory M1 macrophages into anti-inflammatory M2 phenotypes through signaling molecules like interleukin-10 (IL-10) and transforming growth factor-beta (TGF-β) (Murray et al., 2014). This shift plays a critical role in autoimmune conditions such as multiple sclerosis, where MSCs also promote the expansion of regulatory T cells (Tregs) to enhance immune tolerance (Hu et al., 2024).

In addition to their immunomodulatory effects, MSCs secrete a wide array of trophic factors that support tissue repair. Their secretome contains growth factors, chemokines, and EVs that collectively foster regeneration. For example, vascular endothelial growth factor (VEGF) and basic fibroblast growth factor (bFGF) promote new blood vessel formation, improving perfusion to injured areas (Han et al., 2022; Patel et al., 2021). Hepatocyte growth factor (HGF) contributes to antifibrotic effects by limiting collagen accumulation in organs like the liver and lungs (Dohi et al., 2000; Kwiecinski et al., 2011). Meanwhile, insulin-like growth factor 1 (IGF-1) and stromal-derived factor-1 (SDF-1) play protective roles by inhibiting cell death and preserving tissue structure (Al-Samerria and Radovick, 2021; Heo et al., 2019). These diverse and complementary mechanisms highlight the broad therapeutic potential of MSCs across a range of diseases and injury models.

### 1.2 Mitochondrial transfer: a novel mechanism

Recent research has uncovered an innovative mechanism by which mesenchymal stem cells (MSCs) facilitate tissue repair: the transfer of mitochondria (Li et al., 2019). Through the development of tunneling nanotubes slender, dynamic membrane structures MSCs can deliver healthy mitochondria directly to damaged cells, thereby restoring cellular energy production in compromised tissues (Delage et al., 2016; Luchetti et al., 2022). This mechanism has shown significant potential in conditions characterized by mitochondrial dysfunction, such as acute respiratory distress syndrome (ARDS) and myocardial ischemia (Lee et al., 2024; Lesnefsky et al., 2017).

In ARDS, MSCs have been observed to transfer mitochondria to alveolar epithelial cells, resulting in increased ATP generation, decreased oxidative stress, and improved survival outcomes in preclinical models (Wang et al., 2025). Similarly, in the context of myocardial ischemia, mitochondrial transfer to cardiomyocytes helps counteract ischemia-reperfusion injury by stabilizing mitochondrial membrane potential and reducing cell death (Xu et al., 2025). This novel mechanism underscores the adaptive capabilities of MSCs and broadens their therapeutic scope beyond traditional paracrine signaling, offering a promising new avenue for treating diseases marked by impaired cellular energetics.

### 1.3 Therapeutic applications of MSCs

The broad therapeutic potential of mesenchymal stem cells (MSCs) is reflected in their effectiveness across a wide range of clinical conditions. In autoimmune and inflammatory diseases, MSCs have shown significant clinical benefit (Zaripova et al., 2023). For example, in a phase III trial of Remestemcel-L, an MSC product derived from bone marrow, infusions markedly alleviated symptoms in pediatric patients with steroid-refractory acute GVHD, with an overall response rate of 70.4% at day 28 and durable benefit (Kurtzberg et al., 2020). In cases of treatment-resistant rheumatoid arthritis (RA), intra-articular injections of MSCs have been found to reduce synovial inflammation and promote cartilage regeneration (Lopez-Santalla et al., 2020). Similarly, preclinical studies on inflammatory bowel disease (IBD) highlight the ability of MSCs to modulate immune responses (Saadh et al., 2024); MSC treatment reduces colitis severity by inducing macrophage polarization toward an anti-inflammatory state through the secretion of interleukin-10 (IL-10) (Hosseini-Asl et al., 2020; Shi et al., 2019).

In neurological disorders, MSCs offer unique therapeutic advantages due to their capacity to cross the blood-brain barrier and release neuroprotective factors. For instance, MSC-derived exosomes have been shown to slow motor neuron degeneration in animal models of amyotrophic lateral sclerosis (ALS) (Gschwendtberger et al., 2023), and ongoing clinical trials such as MASTERS-2 are investigating intravenous MSC therapy to promote neurogenesis and angiogenesis in stroke patients (Li et al., 2021). In the realm of cardiovascular medicine, MSCs also play a pivotal role (Bagno et al., 2018). Studies like the PARACCT trial report that allogeneic MSCs help reduce scar formation and enhance ejection fraction in patients recovering from myocardial infarction (MI) (Jackson et al., 2012). Furthermore, MSC-secreted factors contribute to the attenuation of adverse ventricular remodeling in heart failure, helping to maintain cardiac function (Guan et al., 2025; Ranganath et al., 2012).

The COVID-19 pandemic underscored the therapeutic relevance of MSCs in acute respiratory conditions. Clinical trials such as REMEDY demonstrated that umbilical cord-derived MSCs (UC-MSCs) could lower mortality rates and improve oxygenation in patients with severe COVID-19 by suppressing cytokine storms and supporting lung tissue repair (Can and Coskun, 2020; Li et al., 2025). These examples collectively emphasize the wide-ranging clinical applications of MSCs, driven by their immunomodulatory, regenerative, and protective capabilities.

### 1.4 Advantages over other stem cell types

Mesenchymal stem cells (MSCs) offer several key advantages over embryonic stem cells (ESCs) and induced pluripotent stem cells (iPSCs), making them particularly well-suited for clinical use. From an ethical standpoint, MSCs avoid the controversies linked to ESCs, as their isolation does not involve the destruction of embryos (Lo and Parham, 2009). In terms of safety, MSCs present a significantly lower risk of tumor formation, especially when compared to the teratoma-forming potential of pluripotent stem cells (Lee and Hong, 2017). Moreover, the establishment of allogeneic MSC banks allows for readily available, “off-the-shelf” therapeutic products, enabling rapid intervention in urgent clinical scenarios such as acute respiratory distress syndrome (ARDS) or myocardial infarction (Garcia-Bernal et al., 2021). These practical advantages coupled with MSCs’ robust immunomodulatory and regenerative functions make them a compelling and scalable option for widespread therapeutic applications (Patel et al., 2013).

### 1.5 Challenges and controversies

Despite their therapeutic potential MSC therapies face several critical challenges that hinder clinical translation. One major obstacle is the variability in MSC potency, influenced by factors such as donor age, tissue origin (e.g., bone marrow vs adipose tissue), and *in vitro* culture conditions (Zhou et al., 2021). This heterogeneity complicates standardization and leads to inconsistent therapeutic outcomes. Another limitation is the poor engraftment efficiency of MSCs; studies indicate that less than 5% of intravenously administered cells successfully home to and persist in target tissues (Burdick et al., 2016). As a result, there is a growing need for strategies that enhance MSC trafficking and retention at injury sites (Ullah et al., 2019).

Clinical trial results have also been mixed. While MSC-based treatments have shown encouraging outcomes in conditions like graft-versus-host disease (GVHD) (Kadri et al., 2023) and severe COVID-19, trials for other diseases such as chronic obstructive pulmonary disease (COPD) have failed to achieve primary endpoints (Broekman et al., 2018). These inconsistencies highlight the importance of developing biomarker-guided approaches to better identify patients who are most likely to benefit and refining delivery methods to improve efficacy. Bridging the gap between preclinical promise and clinical success will require deeper mechanistic insights and innovative bioengineering solutions to fully realize the therapeutic capabilities of MSCs.

## 2 Mechanistic insights: how MSCs exert their effects in vivo

MSCs exhibit remarkable therapeutic potential through a diverse array of biological mechanisms that allow them to repair injured tissues, modulate immune responses, and restore homeostasis across various disease conditions (Jimenez-Puerta et al., 2020). While early research emphasized their capacity for direct differentiation into tissue-specific cell types, contemporary studies reveal that MSCs primarily act through paracrine signaling, immunomodulation, and novel cell-to-cell interactions such as mitochondrial transfer (Velarde et al., 2022). These mechanisms operate synergistically, allowing MSCs to adapt dynamically to injury microenvironments, even in the absence of long-term engraftment. Below, we delve into the molecular and cellular pathways underpinning MSC functionality, integrating preclinical discoveries with clinical evidence to illustrate their translational relevance.

### 2.1 Paracrine signaling: the secretome as a driver of regeneration

The paracrine activity of MSCs mediated by their secretome, a rich cocktail of EVs, growth factors, cytokines, and chemokines, it is now recognized as the cornerstone of their therapeutic effects (Gonzalez-Gonzalez et al., 2020; Ulpiano et al., 2023; Malhotra et al., 2022) Figure 2. Unlike terminally differentiated cells, MSCs secrete bioactive molecules that act on neighboring cells to promote angiogenesis, suppress apoptosis, and mitigate fibrosis, creating a regenerative niche conducive to healing (Gong et al., 2017; Nazarie Ignat et al., 2021; Zou et al., 2023). For instance, vascular endothelial growth factor (VEGF) and basic fibroblast growth factor (bFGF) secreted by MSCs stimulate endothelial cell proliferation and blood vessel formation, a process critical for revascularization ischemic tissues in conditions such as myocardial infarction (MI) and diabetic ulcers (Patel et al., 2023; Patel et al., 2021; Potapova et al., 2007). In the landmark PARACCT trial, allogeneic MSC administration in MI patients reduced infarct scar size by 33% and improved left ventricular ejection fraction, outcomes attributed in part to VEGF-driven angiogenesis (Natsumeda et al., 2017; Williams et al., 2013).

**FIGURE 2:**
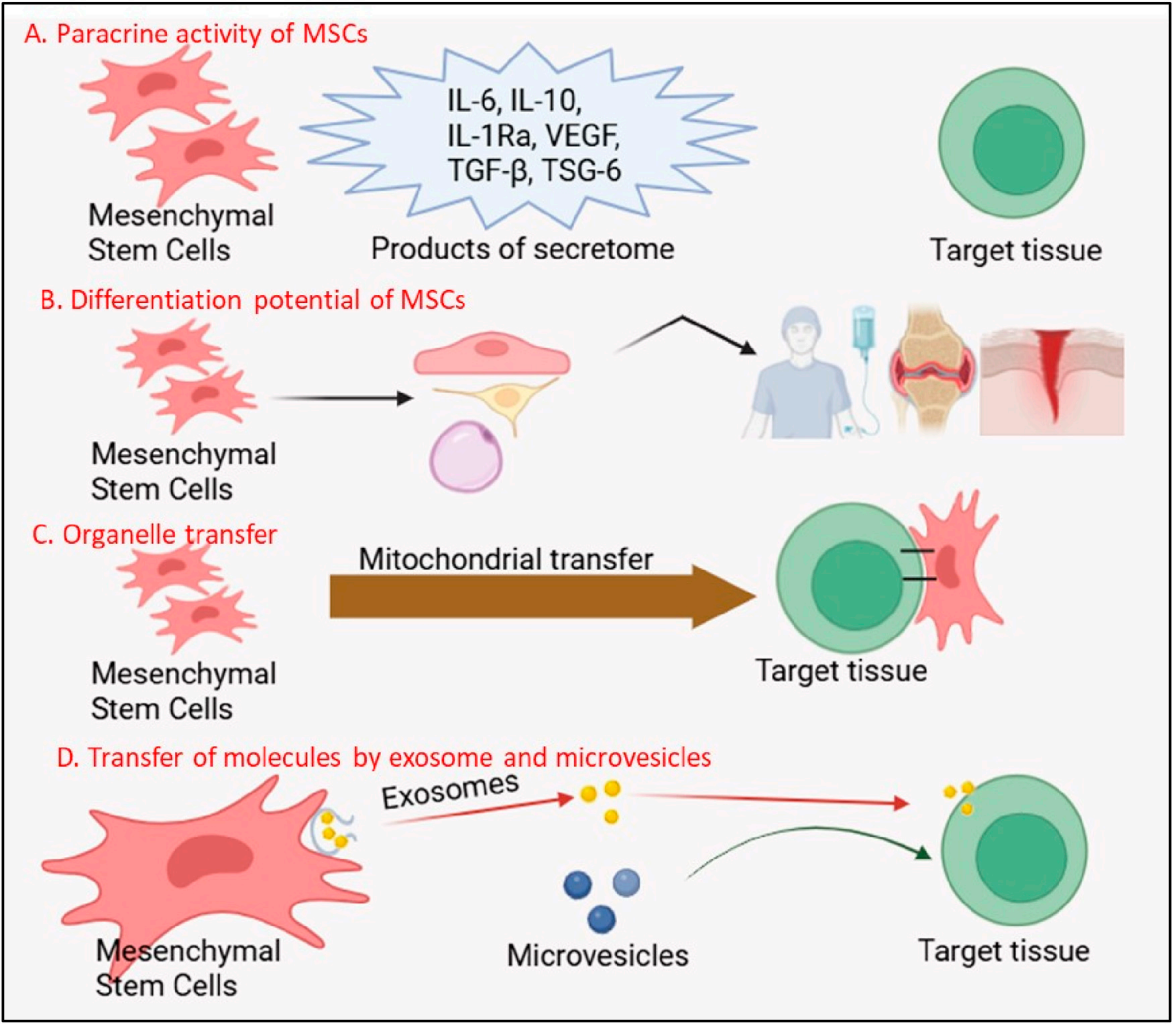
Multifunctional Mechanisms of Mesenchymal Stem Cells (MSCs) in Regenerative Therapy. The figure illustrates four key mechanisms through which MSCs exert their therapeutic effects: **(A)** Paracrine Activity of MSCs: MSCs secrete a variety of bioactive molecules, including anti-inflammatory cytokines (IL-6, IL-10, IL-1Ra), growth factors (VEGF, TGF-β), and tissue-protective factors (TSG-6), which modulate immune responses and promote tissue repair. **(B)** Differentiation Potential of MSCs: MSCs possess the ability to differentiate into multiple cell lineages (e.g., osteocytes, chondrocytes, adipocytes), contributing directly to tissue regeneration. The secretome of MSCs further supports this process by creating a regenerative microenvironment. **(C)** Organelle Transfer: MSCs can transfer functional organelles, such as mitochondria, to damaged cells, restoring cellular metabolism and enhancing survival in target tissues. **(D)** Transfer of Molecules by Exosomes and Microvesicles: MSCs release exosomes and microvesicles containing proteins, nucleic acids, and signaling molecules, which are taken up by recipient cells to mediate therapeutic effects, including immune modulation and tissue repair.

Extracellular vesicles, particularly exosomes, are pivotal mediators of MSC paracrine effects Figure 2. These nanosized lipid bilayer vesicles carry proteins, lipids, and nucleic acids (e.g., microRNAs, mRNAs) that reprogram recipient cells. In neurological disorders like amyotrophic lateral sclerosis (ALS), MSC-derived exosomes deliver neuroprotective miRNAs (e.g., miR-21-5p) to motor neurons, inhibiting pro-apoptotic pathways and delaying disease progression in rodent models (Chen et al., 2021; Malhotra et al., 2022). Similarly, in acute kidney injury, MSC exosomes enriched with miR-30c-5p suppress mitochondrial fission and oxidative stress, preserving renal function (Tsuji et al., 2023). The MASTERS-2 clinical trial, which investigates intravenous MSC therapy for ischemic stroke, has identified exosomal miR-124 as a key mediator of neurogenesis and angiogenesis, bridging preclinical findings to human applications (Yang et al., 2017).

The anti-fibrotic and anti-apoptotic properties of the MSC secretome further underscore its therapeutic versatility (Alfarano et al., 2012; Giacomini et al., 2023). Hepatocyte growth factor (HGF) secreted by MSCs inhibits TGF-β1-driven collagen deposition in fibrotic liver and lung tissues (Wei et al., 2021), while insulin-like growth factor 1 (IGF-1) and stromal-derived factor-1 (SDF-1) activate survival pathways in cardiomyocytes and neurons, respectively (Baglio et al., 2012; Park et al., 2016). In idiopathic pulmonary fibrosis (IPF), MSC-conditioned media reduces myofibroblast activation and collagen synthesis in preclinical models, prompting ongoing Phase II trials (e.g., AETHER-1) exploring aerosolized MSC secretions as a non-cell-based therapy (Filidou et al., 2022).

### 2.2 Immunomodulation: orchestrating immune homeostasis

MSCs possess a unique ability to dynamically modulate immune responses, balancing pro-inflammatory and anti-inflammatory signals to restore homeostasis (Faria et al., 2023). This immunomodulatory capacity is context-dependent: MSCs suppress hyperactive immune reactions in autoimmune diseases while enhancing pathogen clearance in infections (Chen et al., 2024). A key mechanism is their interaction with T cells. By secreting prostaglandin E2 (PGE2) and indoleamine 2,3-dioxygenase (IDO), MSCs inhibit T-cell proliferation and shift the Th1/Th17-Th2/Treg balance toward tolerance (Terraza-Aguirre et al., 2020). In graft-versus-host disease (GVHD), a life-threatening complication of hematopoietic stem cell transplantation, MSC infusions reduce pro-inflammatory cytokines (e.g., IFN-γ, IL-17) and expand regulatory T cells (Tregs), as demonstrated in the Phase III REMODEL trial, where 60% of steroid-refractory GVHD patients achieved complete remission (Kebriaei et al., 2020).

Macrophage polarization represents another critical axis of MSC-mediated immunomodulation. MSCs reprogram pro-inflammatory M1 macrophages into anti-inflammatory M2 phenotypes via interleukin-10 (IL-10) and transforming growth factor-beta (TGF-β) (Cao et al., 2010; Lu et al., 2023). In inflammatory bowel disease (IBD), this shift reduces colonic inflammation (Saadh et al., 2023) and promotes mucosal healing, as evidenced by decreased TNF-α and increased IL-10 levels in murine colitis models (Jung et al., 2015). Clinical trials in Crohn’s disease patients (e.g., Cx601 trial) have shown that locally administered MSCs induce fistula closure through macrophage reprogramming, highlighting translational success (Molendijk et al., 2015).

MSCs also interact with dendritic cells (DCs) and natural killer (NK) cells to fine-tune immunity. By inhibiting DC maturation and antigen presentation via cell-cell contact and soluble factors (e.g., galectin-3), MSCs prevent excessive T-cell activation (Duffy et al., 2011; Simovic Markovic et al., 2016; Sioud et al., 2010; Spaggiari et al., 2009). Simultaneously, they suppress NK cell cytotoxicity by downregulating activating receptors (e.g., NKG2D), reducing tissue damage in conditions like rheumatoid arthritis (RA) (Dehnavi et al., 2023). Intra-articular MSC injections in RA patients have reduced synovial inflammation and cartilage degradation, correlating with diminished NK cell activity and IL-6 levels in synovial fluid (Augello et al., 2007; Papadopoulou et al., 2012).

### 2.3 Mitochondrial transfer and beyond: direct cellular rejuvenation

Emerging research has unveiled a groundbreaking mechanism by which MSCs directly rejuvenate injured cells: mitochondrial transfer Figure 2. Through tunneling nanotubes dynamic membrane channels connecting cells MSCs donate functional mitochondria to energy-depleted cells, restoring ATP production and mitigating oxidative stress (Luchetti et al., 2022). In acute respiratory distress syndrome (ARDS), MSC-derived mitochondria integrate into alveolar epithelial cells, rescuing them from apoptosis and improving gas exchange in preclinical models (Su et al., 2021). A randomized controlled trial conducted in Indonesia evaluated umbilical cord-derived mesenchymal stromal cells (UC-MSCs) as an adjuvant therapy for critically ill COVID-19 patients and demonstrated a 2.5-fold increase in survival, likely due to immunomodulatory effects such as reduced interleukin-6 levels (Dilogo et al., 2021).

Mitochondrial transfer also plays a pivotal role in cardiovascular repair. In myocardial ischemia-reperfusion injury, MSCs donate mitochondria to cardiomyocytes, preserving mitochondrial membrane potential and reducing infarct size by 40% in rodent studies (Huang et al., 2021). This process is enhanced under hypoxic conditions, which upregulate MSC TNT formation. Beyond mitochondria, MSCs transfer lysosomes to cells with defective autophagy, as seen in neurodegenerative diseases like Parkinson’s, where lysosomal delivery clears α-synuclein aggregates and restores neuronal health (Shin and Lee, 2020).

Complementing these direct interactions, MSC-derived extracellular vesicles carry mitochondrial components (e.g., mitochondrial DNA, proteins) that independently boost cellular bioenergetics. In stroke models, MSC-EVs enriched with mitochondrial cytochrome c oxidase enhance neuronal survival (Hermann et al., 2025), while in aging-related osteoporosis, mitochondrial tRNA transfers from MSCs rejuvenate osteoblast function (Jia et al., 2020). These findings underscore the adaptability of MSCs, which employ both secretory and direct contact-dependent strategies to address diverse pathologies.

### 2.4 Integration of mechanisms and clinical translation

The therapeutic efficacy of MSCs *in vivo* arises from the synergistic integration of paracrine, immunomodulatory, and direct cellular mechanisms (Fontaine et al., 2016). For example, in COVID-19-associated ARDS, MSCs concurrently mitigate cytokine storms (via PGE2 and IDO) (Zhang et al., 2022), promote lung vascular repair (via VEGF), and rejuvenate alveolar cells (via mitochondrial transfer) (Tunstead et al., 2024), as evidenced by improved oxygenation and reduced mortality in clinical trials. Similarly, in heart failure, MSC secretome factors (e.g., SDF-1) recruit endogenous stem cells, while mitochondrial transfer enhances cardiomyocyte survival, collectively improving cardiac output (Chung et al., 2015).

However, challenges persist in optimizing MSC homing, survival, and engraftment. Less than 5% of systemically administered MSCs reach target tissues due to pulmonary sequestration and anoikis (Gorshkova et al., 1996; Masterson et al., 2021). Innovations such as magnetic nanoparticle labeling, hypoxia preconditioning, and 3D bio printed scaffolds enhance MSC retention and potency (De Palma et al., 2025). For instance, MSCs preconditioned with TNF-α exhibit upregulated CXCR4 expression, improving homing to ischemic myocardium (Ziaei et al., 2014).

The mechanistic diversity of MSCs spanning paracrine signaling, immune regulation, and direct cellular rejuvenation positions them as versatile therapeutic agents. By leveraging these pathways through bioengineering and targeted delivery, researchers can unlock their full potential, bridging the gap between preclinical promise and clinical reality.

## 3 Preclinical evidence: animal models, efficacy, and safety profiling

The preclinical evaluation of mesenchymal stem cells (MSCs) in animal models has been instrumental in validating their therapeutic potential, elucidating mechanisms of action, and establishing safety profiles prior to human trials (Lee et al., 2014). These studies span a wide array of diseases, leveraging rodents, rabbits, pigs, and non-human primates to mimic human pathologies (Feng et al., 2014; Liu et al., 2016; Pelizzo et al., 2015; Xun et al., 2022). While preclinical data have consistently highlighted the efficacy of MSCs in mitigating tissue damage, modulating immune responses, and promoting regeneration (Cao et al., 2015; Dimarino et al., 2013; Schafer et al., 2016), they also expose critical limitations and translational gaps that complicate the extrapolation of results to clinical settings (Caplan et al., 2019; Levy et al., 2020). This section synthesizes disease-specific preclinical applications of MSCs, underscores their successes, and interrogates the challenges inherent in animal models that hinder seamless translation to human therapies.

### 3.1 Disease-specific applications in preclinical studies

Preclinical research has demonstrated the versatility of MSCs across diverse disease domains, including cardiovascular, neurological, autoimmune, and degenerative disorders. In cardiovascular diseases, rodent models of myocardial infarction (MI) have been pivotal in establishing MSC efficacy (Zhou et al., 2021). For instance, intramyocardial injection of bone marrow-derived MSCs (BM-MSCs) in rats reduced infarct size by 30%–40%, improved left ventricular ejection fraction, and enhanced angiogenesis through secretion of vascular endothelial growth factor (VEGF) and stromal-derived factor-1 (SDF-1) (Poomani et al., 2022). Similarly, porcine models of ischemia-reperfusion injury revealed that MSC therapy attenuated ventricular remodeling and fibrosis, with adipose-derived MSCs (AD-MSCs) outperforming BM-MSCs in promoting cardiomyocyte survival due to their higher angiogenic cytokine output (Gubert et al., 2021; Hegde et al., 2024).

In neurological disorders, MSC efficacy has been explored in rodent models of stroke, traumatic brain injury (TBI), and neurodegenerative diseases (van Velthoven et al., 2017). Intravenously administered MSCs in middle cerebral artery occlusion (MCAO) mice migrated to ischemic brain regions, secreted brain-derived neurotrophic factor (BDNF), and reduced infarct volume by 50%, correlating with improved motor function (Jeong et al., 2014). In Alzheimer’s disease (AD) transgenic mice, MSC-derived exosomes carrying miR-29c-3p suppressed β-amyloid aggregation and neuroinflammation, delaying cognitive decline (Sha et al., 2021). Studies using Parkinson’s disease models have shown that intranasally administered MSCs can cross the blood-brain barrier, reduce dopaminergic neuron loss, and improve motor coordination through mechanisms involving mitochondrial transfer and modulation of glial cell activity.

Autoimmune and inflammatory diseases have also been a focus, with murine models of multiple sclerosis (experimental autoimmune encephalomyelitis, EAE) and rheumatoid arthritis (collagen-induced arthritis, CIA) showcasing MSC immunomodulatory prowess (Constantinescu et al., 2011; Lopez-Santalla et al., 2021). In EAE mice, systemic MSC administration reduced demyelination and Th17-mediated inflammation by expanding regulatory T cells (Tregs) and suppressing dendritic cell activation. CIA models demonstrated that intra-articular MSC injections lowered synovial IL-6 and TNF-α levels, while promoting cartilage repair through chondrocyte differentiation (Lu et al., 2016). Notably, MSC therapy in a lupus-prone (MRL/lpr) mouse model extended survival by curtailing autoantibody production and renal inflammation, findings that informed subsequent Phase I/II trials in systemic lupus erythematosus (SLE) patients (Guo et al., 2023).

Pulmonary diseases, including idiopathic pulmonary fibrosis (IPF) and acute respiratory distress syndrome (ARDS), have benefited from preclinical MSC studies (Yuan et al., 2024). Bleomycin-induced pulmonary fibrosis in mice revealed that MSC-derived hepatocyte growth factor (HGF) suppressed TGF-β1-driven collagen deposition and myofibroblast activation, improving lung compliance (Shukla et al., 2009). In LPS-induced ARDS models, MSCs attenuated alveolar edema and neutrophil infiltration via prostaglandin E2 (PGE2)-mediated macrophage polarization to the M2 phenotype (Hezam et al., 2023). These outcomes were replicated in porcine models of ventilator-induced lung injury, where MSC therapy reduced systemic cytokine storms and improved oxygenation, laying the groundwork for COVID-19 clinical trials.

Orthopedic applications of MSCs, particularly in bone and cartilage repair, have been validated in large animal models. Ovine osteochondral defect studies demonstrated that MSC-loaded scaffolds enhanced hyaline cartilage regeneration and subchondral bone integration, outperforming microfracture techniques (Araki et al., 2015; Wang et al., 2024). Similarly, canine models of spinal cord injury showed that MSC-seeded hydrogels promoted axonal regrowth and functional recovery, with MRI evidence of reduced lesion volume (Cai et al., 2023; Straley et al., 2010).

### 3.2 Limitations and translational gaps in animal models

Despite promising preclinical outcomes, the translation of MSC therapies to the clinic remains hampered by several key limitations. One major challenge is the discrepancy in disease pathophysiology between animal models and human conditions. For instance, bleomycin-induced lung fibrosis in mice does not replicate the chronic, multifactorial progression of IPF in humans, which involves complex genetic and environmental interactions (Lawson et al., 2013). Similarly, rodent stroke models often lack relevant comorbidities such as hypertension or diabetes, leading to overly optimistic assessments of therapeutic efficacy (Macrae, 2011). The use of young, genetically uniform animals further limits clinical relevance, as human patients are typically older and biologically diverse, with diminished regenerative potential (Osier et al., 2016)

Species-specific immune responses also hinder translation. Murine macrophages display distinct cytokine profiles and polarization behaviors compared to human cells, often exaggerating MSCs anti-inflammatory effects (Chen et al., 2023). While humanized mouse models improve immunological relevance, they are expensive and still do not fully replicate human immune complexity (Chuprin et al., 2023). Additionally, although MSCs are considered immune-privileged in rodents, clinical studies show they may be recognized and cleared in immunocompetent humans, reducing their long-term effectiveness (Ankrum et al., 2014).

Technical inconsistencies further complicate translation. Variability in MSC sourcing, donor age, culture protocols, and delivery methods affects cell behavior and therapeutic outcomes (Heyman et al., 2025; Kolliopoulos et al., 2023). For example, intravenous MSCs often become sequestered in the lungs, while local injections improve tissue retention but are less applicable for systemic diseases (Sanchez-Diaz et al., 2021). Most preclinical studies also lack long-term follow-up, missing delayed adverse effects such as tumorigenicity. Rare cases of MSC-induced osteosarcoma in immunodeficient mice raise safety concerns, especially in immunosuppressed patients (Christodoulou et al., 2018). Finally, dose scaling from small animals to humans remains imprecise, with large-animal studies underutilized due to high costs. To bridge these gaps, researchers are adopting humanized models, organ-on-chip platforms, and machine learning to better simulate human physiology and refine preclinical study designs (Ching et al., 2021; Ingber, 2022).

## 4 Clinical translation: progress and pitfalls in human trials

The development of MSC therapies has been characterized by both promising breakthroughs and significant hurdles. Over the past 2 decades, numerous clinical trials have explored the application of MSCs across a broad spectrum of diseases including autoimmune, degenerative, inflammatory, and ischemic conditions providing valuable insights into their safety, therapeutic potential, and translational limitations (Hussen et al., 2024; Patel et al., 2013). Early-phase studies (Phase I/II) have largely confirmed the safety and feasibility of MSC administration, with preliminary evidence supporting their clinical benefit in select cases (Harris et al., 2018). However, as trials have advanced to Phase III, challenges related to standardization, scalability, and reproducibility have become increasingly evident, particularly under heightened regulatory oversight (Taylor et al., 2022). These issues highlight the inherent complexities of translating MSC-based therapies into routine clinical use. This section reviews the progress made in human trials, distills lessons learned from early and late-stage studies, and examines the key barriers that must be overcome to fully realize the clinical potential of MSC therapies (Hetta et al., 2025; da Silva et al., 2025).

### 4.1 Phase I/II trials: safety and early efficacy

Phase I and II trials represent critical early stages in the clinical translation of MSC therapies, focusing primarily on safety while offering initial insights into efficacy. Globally, over 1,200 MSC-related trials have been registered, the majority in these early phases (Galderisi et al., 2022; Guan et al., 2025). Across diverse delivery routes intravenous, intra-articular, intramyocardial, and intrathecal MSC therapies have shown consistent short-term safety, with minimal adverse effects (Bagno et al., 2022). For instance, intravenous infusion of BM-MSCs in GVHD patients at doses up to 10 million cells/kg produced no acute toxicity or ectopic tissue formation. Likewise, intra-articular injections of AD-MSCs in osteoarthritis were well-tolerated, with only transient swelling (Song et al., 2020).

These trials also provide compelling early evidence of efficacy. A pivotal Phase II study (NCT00366145) in steroid-refractory GVHD reported a 70% response rate following BM-MSC infusion, prompting further validation in the Phase III REMODEL trial (Kebriaei et al., 2020). In Crohn’s disease-related fistulas, the Cx601 trial showed a 50% closure rate with local AD-MSC administration, leading to EMA approval of Alofisel in 2018 (Panes et al., 2018). The POSEIDON trial for ischemic cardiomyopathy found that allogeneic MSCs improved ejection fraction and reduced scar size. In neurology, MASTERS-2 demonstrated improved stroke outcomes and MRI evidence of white matter repair, while MSCs slowed disease progression in ALS and reduced mortality in severe COVID-19 cases (Hare et al., 2017).

However, key challenges persist. Product variability due to donor age, tissue source, and culture conditions continues to affect consistency. Optimal dosing and delivery remain unresolved, as intravenous MSCs are largely sequestered in the lungs. Moreover, patient selection is often suboptimal, with few trials stratifying based on biomarkers of response. These limitations underscore the need for more precise trial design to enhance reproducibility and therapeutic impact.

### 4.2 Phase III trials and regulatory challenges

Phase III trials serve as the definitive evaluation of MSC therapies, demanding robust evidence of efficacy, safety, and clinical benefit compared to standard care. While several MSC products have progressed to this stage, results have been inconsistent, reflecting the complexities of large-scale translation (Parekkadan and Milwid, 2010; Cesnik and Svajger, 2024).

In acute GVHD, the Phase III REMODEL trial (NCT02336230) achieved a 60% complete response rate with BM-MSCs, leading to the approval of Temcell in Japan in 2015 the first globally sanctioned allogeneic MSC product (Kurtzberg et al., 2020; Galipeau, 2020). In contrast, the Phase III STAR trial for chronic GVHD failed to meet its primary endpoint, underscoring disease-specific differences in MSC responsiveness (Kadri et al., 2023). Similarly, the ADMIRE-CD trial demonstrated a 53% fistula closure rate in Crohn’s disease patients, securing EMA approval for Alofisel, though the FDA withheld approval due to concerns over long-term durability (Garcia-Olmo et al., 2022).

Cardiovascular trials have proven more challenging. The CHART-1 trial in chronic heart failure showed neutral results despite promising earlier data, likely due to variability in cell potency and delivery (Rheault-Henry et al., 2021; Tompkins et al., 2017). The CHART-1 trial utilized cardiopoietic MSCs that underwent lineage-specific induction, representing a differentiated MSC therapy rather than native MSC administration (Dilogo et al., 2021). The TRIDENT trial also failed to demonstrate significant improvement in myocardial infarction (Piccini et al., 2011). Neurological studies faced similar setbacks; the NeuroNEXT ALS trial showed no survival benefit, and MASTERS-2 revealed reduced effect sizes in Phase III, reflecting challenges in scaling early success to broader populations (Cudkowicz et al., 2022; Shefner and Cudkowicz, 2024).

These discrepancies underscore systemic issues in late-stage MSC development:

a. Product Standardization: Unlike pharmaceuticals, MSCs are live products sensitive to manufacturing variables. The absence of universal potency assays metrics quantifying MSC function (e.g., immunosuppressive capacity, VEGF secretion) has led to batch-to-batch variability. For example, Prochymal, an MSC product for GVHD, faced criticism for inconsistent IDO enzyme activity across batches, potentially undermining efficacy (Chinnadurai et al., 2018).
b. Placebo Effects and Trial Design: Many MSC trials lack adequate blinding, particularly in open-label surgical deliveries (e.g., intramyocardial injections), inflating placebo effects. The ACT34-CMI trial for critical limb ischemia was confounded by high placebo responses, obscuring MSC benefits (Losordo et al., 2011; Losordo et al., 2012).
c. Regulatory Fragmentation: Global regulatory agencies impose conflicting requirements. The EMA emphasizes long-term safety and immunogenicity data, while the FDA prioritizes mechanistic biomarkers and potency assays (Kang et al., 2023). Japan’s PMDA, meanwhile, fast-tracks MSC approvals based on Phase II data for unmet needs, creating uneven commercial landscapes.

To overcome manufacturing and regulatory challenges, MSC innovators are embracing advanced technologies such as closed-system bioreactors and xenogen-free culture media to enhance consistency, while improved cryopreservation extends shelf life without compromising cell viability (Rogers et al., 2021). Trials like MSC-NTF in ALS have demonstrated the benefits of potency standardization through neurotrophic factor enrichment. Regulatory agencies are also evolving; the FDA’s 2022 draft guidance recommends potency assays aligned with mechanism of action, such as IDO activity for immunomodulatory products, promoting better translational alignment (Hoang et al., 2025).

MSC therapies have reached a pivotal stage in clinical translation. While early-phase trials affirm safety and signal therapeutic promise in conditions from GVHD to COVID-19 ARDS, Phase III failures have underscored the need for improved standardization, mechanistic clarity, and precision in trial design (Horie et al., 2020; Ragel et al., 2023). Successes like Alofisel and Temcell show that with robust manufacturing and targeted applications, regulatory approval is attainable (Lu and Allickson, 2024). Future progress hinges on biomarker-guided patient selection, validated potency assays, and adaptive clinical strategies to fully realize the potential of MSCs in regenerative medicine.

## 5 Biosafety and immunogenicity: addressing concerns in MSC therapies

MSC therapies represent a promising frontier in regenerative medicine and immunomodulation, offering therapeutic potential for a wide range of diseases, including osteoarthritis, myocardial infarction, GVHD, and autoimmune disorders (Guo et al., 2020; Mancuso et al., 2019). Their clinical appeal lies in their capacity to differentiate into multiple lineages, secrete trophic factors that promote tissue repair, and modulate immune responses (Ayala-Cuellar et al., 2019). However, as these therapies advance in clinical use, two critical concerns biosafety and immunogenicity must be thoroughly addressed to ensure therapeutic efficacy and patient safety.

Biosafety challenges include risks such as tumorigenicity, unintended differentiation, microbial contamination, genetic instability from extended culture, and issues related to manufacturing consistency (Neri, 2019). Although MSCs are generally considered non-tumorigenic, prolonged *in vitro* expansion may introduce chromosomal abnormalities or oncogenic mutations, increasing the risk of malignant transformation (Momin et al., 2010; Tarte et al., 2010). Rare cases of ectopic tissue formation have been observed in animal studies, though they are infrequent in clinical trials (Fujiwara et al., 2023). To mitigate these risks, regulatory agencies mandate rigorous preclinical testing, including karyotyping, tumorigenicity assays, and genetic stability evaluations via comparative genomic hybridization or next-generation sequencing (Sato et al., 2019). The route of MSC administration also influences safety; while systemic infusion may cause pulmonary entrapment or embolism, localized delivery can limit systemic exposure and off-target effects. Long-term follow-up in clinical trials remains essential for monitoring delayed adverse events.

Immunogenicity, though less severe in MSCs compared to other cell types, presents nuanced challenges, especially in allogeneic applications (Li et al., 2024b). MSCs were once considered immune-privileged due to low MHC class II and co-stimulatory molecule expression (Kapranov et al., 2016). However, repeated dosing or inflammatory environments can prompt immune recognition, especially through HLA mismatches, leading to T-cell or antibody-mediated responses (Ravindranath et al., 2021). MSCs display context-dependent behavior acting as immunosuppressive under certain conditions (e.g., via PGE2 and TGF-β secretion), yet potentially immunogenic when primed by interferon-gamma (IFN-γ) or Toll-like receptor (TLR) activation (Haddad and Saldanha-Araujo, 2014). Strategies to reduce immunogenicity include HLA matching, genetic editing to knock out MHC expression using CRISPR-Cas9, IFN-γ priming to enhance IDO activity, and encapsulation within biomaterials to evade immune surveillance (Hoerster et al., 2020).

MSC source and culture conditions significantly affect both safety and immune compatibility. UC-MSCs often show higher proliferative rates and lower immunogenicity compared to BM-MSCs (Hori et al., 2024). Serum-free media and xenogen-free protocols reduce variability and contamination risks, while 3D and hypoxic cultures help preserve MSC functionality (Gottipamula et al., 2013). Standardized potency assays measuring factors like IDO or PGE2 support batch consistency, while emerging tools such as single-cell RNA sequencing enable deeper characterization of MSC subpopulations with favorable safety profiles (Xie et al., 2022).

Finally, evolving regulatory frameworks are critical to balancing innovation with patient protection. Phase I trials focus on acute toxicity and biosafety, while later stages include immunogenicity monitoring via anti-MSC antibody detection and T-cell assays. Predictive biomarkers, such as soluble HLA-G or extracellular vesicle signatures, are being explored (Shan et al., 2024). As gene-edited or cytokine-engineered MSCs enter trials, regulatory oversight must adapt to assess new risks like insertional mutagenesis or transgene immunogenicity (Jadlowsky et al., 2024). In safeguarding MSC therapy requires an integrated, multidisciplinary approach encompassing advanced manufacturing, precise immunological assessment, and dynamic regulatory adaptation (Fernandez-Santos et al., 2022). Only through such comprehensive strategies can MSCs realize their full clinical potential while maintaining the highest standards of safety and efficacy.

## 6 Scalability and manufacturing: barriers to commercialization

The commercialization of MSC therapies faces major hurdles, including biological variability, regulatory complexity, and challenges in scalable, cost-effective manufacturing (Beheshtizadeh et al., 2022; Zhou et al., 2021). Ensuring product consistency, viability during storage, and compliance with quality standards remains difficult. Overcoming these bottlenecks requires innovations in bioprocessing, automation, and logistics, alongside harmonized regulatory frameworks to streamline development and ensure broad clinical accessibility.

### 6.1 Standardization challenges: donor variability and product heterogeneity

Achieving product consistency is a major barrier to MSC commercialization. Unlike small-molecule drugs, MSCs are living cells whose therapeutic function varies with donor characteristics (e.g., age, sex, health), tissue source (bone marrow, adipose, umbilical cord), and culture conditions (media, oxygen levels) (Mastrolia et al., 2019; Maged et al., 2024). For example, MSCs from older donors exhibit reduced proliferation and diminished secretion of regenerative factors. AD-MSCs produce more VEGF, favoring ischemic indications, while BM-MSCs are often more immunomodulatory (Maged et al., 2024). This biological variability complicates dose standardization and contributes to inconsistent clinical outcomes, as seen in Prochymal’s Phase III failure for GVHD, partly due to variable IDO activity (Kadri et al., 2023). The lack of standardized potency assays further exacerbates the issue. Current ISCT criteria (CD73/CD90/CD105 expression and trilineage differentiation) fail to predict clinical efficacy (Cesnik and Svajger, 2024; Robb et al., 2019). Regulators now recommend mechanism-specific potency tests, e.g., IDO for immunosuppression or VEGF for angiogenesis but integrating these assays into large-scale production remains technically and economically challenging.

### 6.2 Bioprocessing hurdles: from laboratory to industrial scale

Scaling MSC production from lab-scale flasks to industrial-scale bioreactors poses significant technical and economic hurdles (Haskell et al., 2024). Traditional 2D culture systems like T-flasks are labor-intensive, space-consuming, and yield limited cell numbers (∼10^8^ cells per batch), insufficient for clinical demand (Haskell et al., 2024). Microcarrier-based 3D bioreactors offer a 100-fold increase in output but require precise control of variables like shear stress and oxygenation. Systems like Lonza’s Cocoon^®^ automate expansion in closed environments, yet challenges remain in maintaining consistent cell potency aggregates often develop hypoxia-induced senescence (Tang et al., 2025). Cost of goods (COGs) also constrain scalability. GMP-grade, xenogeneic-free media (e.g., human platelet lysate) costs $500–$1,000 per liter, and large-scale doses (100–200 million cells) for indications like myocardial infarction may require $20,000–$50,000 in media alone. Cryopreservation adds further expense, with cold-chain logistics increasing COGs by ∼30% due to the need for ultra-low temperatures (−150°C) and specialized storage systems. Together, these issues complicate the path to commercial viability.

### 6.3 Regulatory and quality control complexities

Navigating regulatory pathways for MSC therapies is complex and inconsistent across regions. Agencies like the FDA and EMA classify MSCs as advanced therapy medicinal products (ATMPs), requiring cGMP compliance, thorough safety evaluations, and detailed manufacturing documentation (Detela and Lodge, 2019). However, divergent guidelines complicate approval, for example, the EMA mandates 24-month tumorigenicity studies in immunodeficient mice, whereas the FDA emphasizes *in vitro* genomic stability assays (Calvisi et al., 2023). Such differences delay global market access, as seen with Alofisel (darvadstrocel), approved in Europe but still under FDA review pending more long-term data.

QC remains a critical challenge. Standard release criteria focus on viability (>70%), sterility, and surface marker identity, but functional assays like immunosuppressive mixed lymphocyte reactions or angiogenesis tests are rarely performed at scale due to cost and timing. This gap risks releasing subpotent products, exemplified by a Phase II COPD trial where MSCs with low hepatocyte growth factor secretion failed to improve outcomes.

### 6.4 Innovations driving scalable manufacturing

Despite significant challenges, advances in bioprocessing and automation are enabling scalable MSC production. Closed-system bioreactors like the Xpansion^®^ platform facilitate high-density 3D culture, reducing media use by 60% and doubling yields (Song et al., 2024). Synthetic peptide substrates replace traditional coatings, improving reproducibility and reducing batch variability. Emerging lyophilization methods promise to eliminate cold-chain dependence, with early data showing 80% viability post-reconstitution.

Machine learning (ML) is revolutionizing quality control, using AI-driven analysis of omics and secretome data for real-time potency prediction and release testing (Wei et al., 2023). For example, Cellino Biotech’s ML platform cuts production costs by 70%. Concurrently, CRISPR-engineered MSCs with enhanced homing (CXCR4) or anti-inflammatory (IL-10) traits are advancing in trials, potentially lowering dose requirements and costs (Zhou et al., 2021; Hazrati et al., 2022). Overcoming scalability and manufacturing barriers demands collaboration across academia, industry, and regulators to standardize processes, adopt disruptive technologies, and align reimbursement with therapeutic value. Addressing these will enable MSC therapies to transition from niche innovations to accessible mainstream treatments in regenerative medicine.

## 7 Emerging strategies: engineering and enhancing MSC functionality

MSCs hold great therapeutic promise but face hurdles like poor engraftment, limited survival, and functional variability. To overcome these, researchers are developing advanced strategies including CRISPR gene editing, biomaterial scaffolds, and synthetic biology tools to boost MSC regenerative, immunomodulatory, and homing abilities (Abubakar et al., 2023; Christidi et al., 2018). Innovations such as bioengineered exosomes and tailored genetic modifications are enhancing efficacy and specificity, paving the way for more effective, disease-targeted MSC therapies (Cao et al., 2024).

### 7.1 Genetic engineering: precision-enhanced MSCs

Genetic engineering is pivotal in enhancing the therapeutic potential of MSCs. Techniques like CRISPR-Cas9 and lentiviral vectors enable precise gene edits to boost survival, targeting, and secretion of therapeutic molecules (Damasceno et al., 2020). For example, MSCs overexpressing CXCR4 demonstrate improved homing to ischemic tissues, with a Phase I trial in critical limb ischemia showing a 40% perfusion increase (Ullah et al., 2019). IL-10-engineered MSCs have reduced synovial inflammation by 70% in rheumatoid arthritis models (Choi et al., 2008; Li et al., 2024a). Advanced approaches using synthetic biology allow programmable behaviors, such as hypoxia-inducible VEGF expression for myocardial repair. Safety mechanisms like suicide genes (e.g., HSV-TK) allow conditional elimination of MSCs, enhancing clinical control (Kim et al., 2011; Shen et al., 2008). These strategies improve efficacy while addressing safety concerns in MSC-based therapies.

### 7.2 Preconditioning: priming MSCs for enhanced performance

Preconditioning MSCs with biochemical or physical stimuli enhances their therapeutic potential without genetic modification. Hypoxic preconditioning (1%–5% O_2_) simulates ischemic tissue conditions, increasing survival gene expression (e.g., Bcl-2) and angiogenic factors like VEGF and SDF-1 (Schepici et al., 2022; Zhuo et al., 2024). In a porcine myocardial infarction model, hypoxia-treated MSCs improved cardiac function by 50% over normoxic controls (Chen et al., 2018). Cytokine priming with IFN-γ or TNF-α enhances immunomodulatory effects by upregulating IDO and PD-L1, beneficial in GVHD. 3D spheroid culture restores in vivo-like MSC phenotypes, increasing paracrine activity and ECM production (Sarsenova et al., 2022; Elmi et al., 2025). In stroke models, spheroid-derived MSCs tripled BDNF and GDNF secretion, improving neuronal survival by 60%. Mechanical preconditioning further boosts MSC secretomes, supporting tissue repair in tendon and vascular injuries (Cunningham et al., 2018).

### 7.3 Biomaterial scaffolds: guiding MSC integration and retention

Biomaterial scaffolds are transforming MSC therapy by enhancing cell retention, survival, and differentiation through structural and biochemical cues (Zhao et al., 2021). Hydrogels made from hyaluronic acid, collagen, or decellularized ECM provide protective niches and controlled release of therapeutic factors (Saldin et al., 2017). In equine osteoarthritis, MSC-loaded thermoresponsive chitosan hydrogels achieved 80% cartilage defect filling in 12 weeks (Spiller et al., 2011; Atwal et al., 2023). Electrospun nanofibers functionalized with RGD peptides or laminin promote MSC adhesion and alignment, aiding nerve and muscle repair (Amores de Sousa et al., 2020). Advanced 3D-printed scaffolds, such as β-TCP, enable anatomically tailored implantation and showed superior calvarial bone healing in rabbits (Guerrero et al., 2024; Turnbull et al., 2018). Smart scaffolds with embedded sensors or drug reservoirs allow real-time monitoring and condition-responsive factor release, improving outcomes in diabetic wound models through glucose-triggered VEGF secretion (Mirani et al., 2023).

### 7.4 Exosome engineering: harnessing MSC-Derived nanovesicles

MSC-derived exosomes are emerging as promising cell-free therapies, offering regenerative benefits without the risks of cell transplantation (Roszkowski, 2024). However, their natural lack of targeting specificity limits efficacy. To enhance precision, exosomes are engineered with surface ligands like CD47 or RGD and loaded with therapeutic cargo such as siRNA or miRNAs (Kim et al., 2024; Zeng et al., 2023). In glioblastoma, RVG-tagged exosomes delivered miR-124 across the blood-brain barrier, reducing tumor volume by 65% (Galardi et al., 2023) (Aili et al., 2021). Techniques like electroporation improve cargo loading, and clinical trials by companies like Codiak Biosciences are underway.

### 7.5 Combination therapies: synergizing MSCs with drugs or cells

Combining MSCs with pharmacological agents or complementary cell types enhances therapeutic efficacy (Abu-El-Rub et al., 2022). MSCs with anti-inflammatory drugs like tocilizumab or JAK inhibitors show synergistic effects, reducing autoantibodies by 90% in refractory lupus trials (Scott, 2017). In cancer therapy, MSCs engineered to express TRAIL improve chemotherapy by targeting resistant cancer stem cells (Fakiruddin et al., 2018; Minev et al., 2024). Co-transplantation strategies, such as MSCs with endothelial progenitor cells (EPCs) or CAR-T cells, enhance outcomes doubling capillary density in limb ischemia and halving cytokine release syndrome severity in leukemia models (Rossi et al., 2017).

### 7.6 AI and machine learning: optimizing MSC manufacturing

Artificial intelligence is revolutionizing MSC bioprocessing by optimizing culture conditions, donor matching, and therapeutic predictions (Cerbo et al., 2024). Deep learning models identify biomarkers like miR-335-5p for chondrogenic potential and refine bioreactor settings to boost efficiency. AI platforms, such as DeepCell, enhance quality control with 95% accuracy in detecting senescent cells (Nosrati and Nosrati, 2023). Together with genetic engineering and biomaterial advances, AI is driving the development of precision-engineered MSC therapies (Mahima Choudhury et al., 2025). As these innovations converge, scalable and effective MSC products become increasingly feasible, though robust safety and regulatory frameworks remain essential for clinical translation.

## 8 Future directions: personalized medicine and advanced delivery systems

The next frontier in MSC therapy lies in integrating personalized medicine with cutting-edge delivery technologies. As the field moves beyond the conventional “one-size-fits-all” paradigm, advances in genomics, biomaterials, and bioengineering are converging to create tailored MSC treatments that align with individual patient profiles and disease-specific microenvironments. These innovations are poised to overcome long-standing challenges, such as poor engraftment, off-target effects, and inconsistent therapeutic outcomes, paving the way for precision regenerative medicine.

Personalized MSC therapies are being revolutionized through biomarker discovery and multi-omics profiling. By leveraging genomics, transcriptomics, proteomics, and metabolomics, researchers can map MSC heterogeneity with unprecedented granularity. For example, single-cell RNA sequencing has identified MSC subsets with distinct therapeutic potentials, such as PD-L1+ cells for immunosuppression or CXCR4+ cells for tissue homing. This stratification allows clinicians to match MSC profiles with patient biomarkers. A 2023 study found that rheumatoid arthritis patients with high IL-6 levels responded better to IFN-γ-primed MSCs, offering a biomarker-driven approach to preconditioning. Autologous customization, using tools like CRISPR-Cas9, further allows correction of genetic mutations or enhancement of therapeutic genes. Clinical trials using gene-edited MSCs to express dystrophin in Duchenne muscular dystrophy have shown promise. Similarly, iPSC-derived MSCs provide a scalable and patient-specific source, currently under evaluation for disorders like age-related macular degeneration. Rieger et al. demonstrated that the genetic profile of patients with non-ischemic dilated cardiomyopathy significantly influenced responsiveness to MSC therapy, with variant-negative individuals deriving the most benefit. These findings underscore the value of precision medicine in MSC-based interventions (Rieger et al., 2019). Machine learning tools are being employed to predict patient responses and optimize MSC product selection. DeepCell Therapeutics, for instance, developed an AI model that successfully stratified stroke patients based on imaging and molecular profiles, reducing trial failure rates.

Advanced delivery systems are enhancing MSC retention, targeting, and therapeutic duration. Biomaterial-assisted platforms like hydrogels, decellularized ECM, and adhesion molecule-infused scaffolds are improving engraftment and guiding MSC differentiation. 3D and 4D bioprinting technologies enable the construction of precise, vascularized tissues with functional gradients. In goat models, bio printed cartilage patches restored joint function, while shape-memory scaffolds adapted to body contours in pediatric microtia patients. Furthermore, stimuli-responsive carriers offer targeted and controlled release. Magnetic nanoparticle-labeled MSCs have achieved over 90% targeting accuracy, and optogenetically engineered MSCs can secrete VEGF in response to light, improving diabetic ulcer treatment.

Digital health integration and closed-loop systems represent the next step in adaptive MSC therapy. Smart implants with biosensors can monitor MSC viability and tissue regeneration, transmitting data in real time. In spinal cord injury models, graphene-based neural interfaces tracked recovery and activated stimulatory cues to enhance repair. Closed-loop systems, such as glucose-responsive devices, have been adapted to release cytokine-secreting MSCs in inflammatory environments, maintaining disease remission. Genetic circuits responsive to tumor DNA allow MSCs to deliver anti-cancer agents like TRAIL specifically within tumor sites, minimizing systemic exposure.

While MSC therapies have demonstrated immense preclinical promise, clinical success requires overcoming biological, technical, and regulatory hurdles. The fusion of personalized omics data, sophisticated delivery systems, and real-time monitoring is transforming MSC therapy into a precise, patient-tailored modality. As these innovations mature, the vision of safe, scalable, and effective MSC-based treatments is becoming an attainable reality.

## 9 Final synthesis

The gap between preclinical promise and clinical reality for MSC therapies is neither unbridgeable nor inevitable. By embracing standardization, personalization, and innovation, the field can transform MSCs from a promising tool into a mainstay of regenerative medicine. The path forward demands humility to learn from past failures and audacity to pioneer technologies that redefine healing. As these cells navigate the complex journey from bench to bedside, their ultimate success will hinge not just on scientific ingenuity, but on our collective commitment to translating hope into tangible, equitable health outcomes.
